# Pressure Ulcers Risk Assessment According to Nursing Criteria

**DOI:** 10.3390/healthcare10081438

**Published:** 2022-07-31

**Authors:** Eugenio Vera-Salmerón, Emilio Mota-Romero, José Luis Romero-Béjar, Carmen Dominguez-Nogueira, Basilio Gómez-Pozo

**Affiliations:** 1 Centro de Salud Dr. Salvador Caballero de Granada, Distrito Sanitario Granada-Metropolitano, Servicio Andaluz de Salud, 41071 Sevilla, Spain; eugenio.vera.sspa@juntadeandalucia.es (E.V.-S.); emilio.mota.sspa@juntadeandalucia.es (E.M.-R.); 2 Instituto de Investigación Biosanitaria (ibs.GRANADA), 18012 Granada, Spain; jlrbejar@ugr.es (J.L.R.-B.); basilio.gomez.sspa@juntadeandalucia.es (B.G.-P.); 3 Department of Nursing, University of Granada, Avda. Ilustración 60, 18071 Granada, Spain; 4 Department of Statistics and Operations Research, University of Granada, Fuentenueva s/n, 18071 Granada, Spain; 5 Institute of Mathematics, University of Granada (IMAG), Ventanilla 11, 18001 Granada, Spain; 6 Inspección Provincial de Servicios Sanitarios, Delegación Territorial de Granada, Consejería de Salud y Familias de la Junta de Andalucía, 41071 Sevilla, Spain; 7 Unidad de Epidemiología y Promoción de la Salud, Distrito Sanitario Granada-Metropolitano, Servicio Andaluz de Salud, 41071 Sevilla, Spain

**Keywords:** activity, Braden Scale, immobilized patients, logistic regression, mobility, pressure ulcers

## Abstract

Pressure ulcers (PU) represent a health problem with a significant impact on the morbidity and mortality of immobilized patients, and on the quality of life of affected people and their families. Risk assessment of pressure ulcers incidence must be carried out in a structured and comprehensive manner. The Braden Scale is the result of an analysis of risk factors that includes subscales that define exactly what should be interpreted in each one. The healthcare work with evidence-based practice with an objective criterion by the nursing professional is an essential addition for the application of preventive measures. Explanatory models based on the different subscales of Braden Scale purvey an estimation to level changes in the risk of suffering PU. A binary-response logistic regression model, supported by a study with an analytical, observational, longitudinal, and prospective design in the Granada-Metropolitan Primary Healthcare District (DSGM) in Andalusia (Southern Spain), with a sample of 16,215 immobilized status patients, using a Braden Scale log, is performed. A model that includes the mobility and activity scales achieves a correct classification rate of 86% (sensitivity (S) = 87.57%, specificity (SP) = 81.69%, positive predictive value (PPV) = 91.78%, and negative preventive value (NPV) = 73.78%), while if we add the skin moisture subscale to this model, the correct classification rate is 96% (S = 90.74%, SP = 88.83%, PPV = 95.00%, and NPV = 80.42%). The six subscales provide a model with a 99.5% correct classification rate (S = 99.93%, SP = 98.50%, PPV = 99.36%, and NPV = 99.83%). This analysis provides useful information to help predict this risk in this group of patients through objective nursing criteria.

## 1. Introduction

Pressure ulcers (PU) are injuries caused to the skin and/or underlying tissues as a result of continuous pressure on these tissues, or due to the combination with shearing. They are usually located on bony prominences [1]. They have a high average prevalence, both in Europe (10.8%) [2] and in Spain, which stands at 7% in the hospital environment [3] and 4.79% among patients in home care [4]. PU represent a health problem with a significant impact on the morbidity and mortality of immobilized patients and in the quality of life of affected people and their families [5]. Risk assessment of pressure ulcers incidences must be carried out in a structured and comprehensive manner. The Braden Scale for Predicting Pressure Sore Risk allows for early identification of patients at risk of pressure ulcers by assessing six subscales that reflect sensory perception, skin moisture, activity, mobility, friction and shear, and nutritional status [6]. The Braden Scale is a widely used tool among clinicians. This scale has been shown to be a valid predictor of the development of pressure ulcers [7,8], in addition to possessing a better balance between the values of sensitivity and specificity compared to other similar tools [9]. In addition, several studies have assessed the predictive value that the different subscales alone may have for the assessment of PU risk [10,11,12].

Evidence-based nursing healthcare practice or evidence-based nursing (EBN) “is the conscientious, explicit, and judicious use of theory-derived, research-based information in making decisions about care delivery to individuals or groups of patients reflective of individual needs and preferences” [13]. EBN improves the quality and safety of health care for patients, reduces healthcare costs [14], and is an essential addition for the application of preventive measures for PU [15]. In this sense, providing confidence in the objective criteria of the nursing professional based on their experience gives them the possibility of making quick decisions that allow them to anticipate risk situations and/or take preventive measures. Indeed, there are already studies that, in some way, attempt to link risk assessment to one of the subscales of the Braden Scale, mainly the activity and mobility subscales [16,17,18], in order to develop prevention strategies for PU, and thus reduce the workload associated with such a major health burden.

Explanatory models based on a different number of Braden subscales combination purvey an efficient estimation to level changes in the risk of suffering PU, as well as the strength of the levels within these subscales for prognosis in a worsening level of risk of developing a PU. This study’s purpose is to identify groups of subscales that provide efficient classification models, and quantify the effect of each subscale within the model for prognosis at a level of worsening risk of developing pressure ulcers for immobilized patients.

## 2. Patients and Methods

### 2.1. Study Design

A study with an analytical, observational, longitudinal, and prospective design was carried out in the Granada-Metropolitan Primary Healthcare District (DSGM) in Andalusia, Spain, with a sample of immobilized patients.

### 2.2. Participants

The study area within the scope of the DSGM, which is urban–rural–mixed, provides health care to a population of 673,959 people (48.74% men and 51.26% women), representing 72.4% of the total population of Granada province. Its health organization is structured around 36 Basic Health Areas and 73 Socio-Health Centers. The total number of people over 64 years old assigned to the DSGM, according to the Spanish National Statistics Institute (INE), was 114,558, of which the estimate of immobilized patients, according to the Andalusian Health Service Portfolio, was 17,183 people (15% of the population >64 years old). The sample comprised 16,215 immobilized-status patients older than 64 years, with Braden Scale measure recorded. The data was collected from the SIRUPP application that is integrated in the Diraya Health History application from the Andalusian Public Health System. All the immobilized patients registered in SIRUPP were considered. The mean age of the participants was 84.13 years (SD = 9.42), and 69.8% of them were female.

### 2.3. Ethical Considerations

The study was carried out in accordance with the 1975 Declaration of Helsinki [19] and was approved by the Clinical Research Ethics Committee at the Andalusian Public Health System (AP-0086-2016).

### 2.4. Statistical Analysis

Firstly, a graphical exploratory analysis was carried out to identify which variables individually, by pairs, and triplets were able to identify patients at higher risk of PU. Second, various binary-response logistic regression models [20] were used to identify the subscales combinations with higher performance for PU risk prediction. A stepwise forward–backward selection model, without any interactions, was deemed to best fit the records. The goodness-of-fit was compared using the probability ratio test and Stukel’s chi-squared test. Wald’s test was used to evaluate the significance at the population level of the factors that entered the models. The validation of the model was done by calculating the rate of correct classifications. The ROC curve was used to analyze the performance of the models. The strength ratios for every level regarding the adjacent level were achieved, according to the potential variations in the risk subscales studied. The R Statistical Computing Software (version 4.1.1) (https://www.r-project.org/) (Accessed on 27 July 2022) was used for the statistical analysis.

## 3. Results

This section is structured as follows. First, a descriptive analysis of the response and explanatory variables is shown. Then, an exploratory analysis based on graphical outputs allows to probe the power for classification, of being in a risk level (or not) for developing pressure ulcers, by means of individual or certain groups of subscales. Section 3.3 estimates a binary logistic regression model for the risk of PU, based on the six subscales. A detailed analysis of prognostic ability of each subscale is performed. In addition, different measures and graphical outputs of the quality of the model from an inferential, accuracy, and validity point of view are provided. The two following sections estimate, analyze, and validate binary logistic regression models based on the activity and mobility subscales (Section 3.3), and based on activity, mobility, and skin moisture subscales (Section 3.4). These last two models are based on the graphical exploratory analysis performed in Section 3.2.

### 3.1. Sample Description

According to the Braden Scale scores, the individuals were classified into: no risk or risk of developing pressure ulcers. The descriptive analysis of the subscales deemed is shown in Table 1.

### 3.2. Exploratory Analysis for Classification

#### 3.2.1. Univariate Graphical Exploratory Analysis

**Figure 1:**
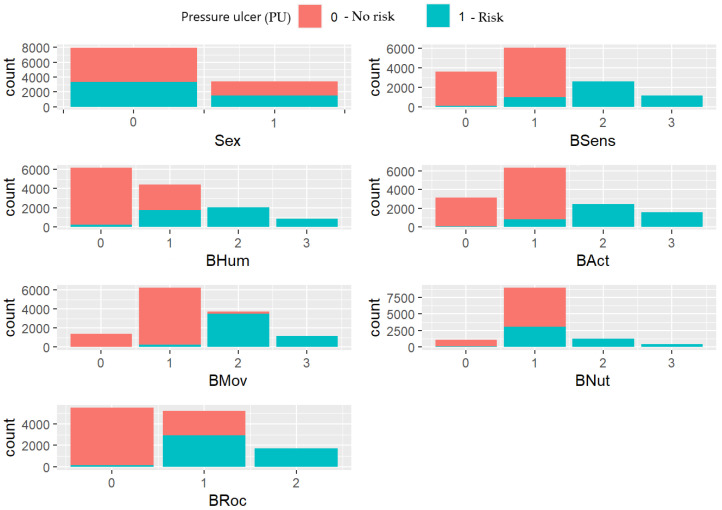
PU risk classification based on individual subscales.

The overlap histograms for the classification of a patient at risk or not of suffering from pressure ulcer, based on sex and each one of the subscales independently, are dis-played in Figure 1. Activity (BAct) and mobility (BMov) subscales seem to be adequate as independent classifiers. This is reflected in their histograms, because the colors that correspond to each level of PU risk are well separated for the different values of these subscales.

#### 3.2.2. Bivariate Graphical Exploratory Analysis

**Figure 2:**
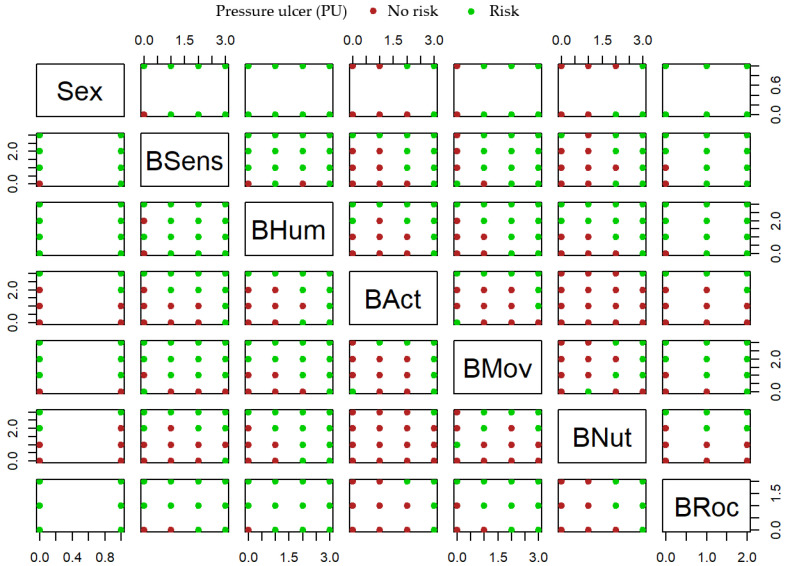
PU risk classification based on two subscales.

Figure 2 shows the potential applicability as classifiers of each pair of variables jointly. In this graphical output it is reflected that activity (BAct) and mobility (BMov) subscales are jointly adequate classifiers because the green and red dots are well separated in the corresponding biplot located (according to a matrix notation) in the 4th row and 5th column. There are more pairs of variables that could be considered jointly as adequate for classification, but keeping in mind that the objective nursing criteria are based on such quick identification and objective information provided by activity and mobility subscales, these are not relevant in this work.

#### 3.2.3. Three-Subscale Graphical Exploratory Analysis

**Figure 3:**
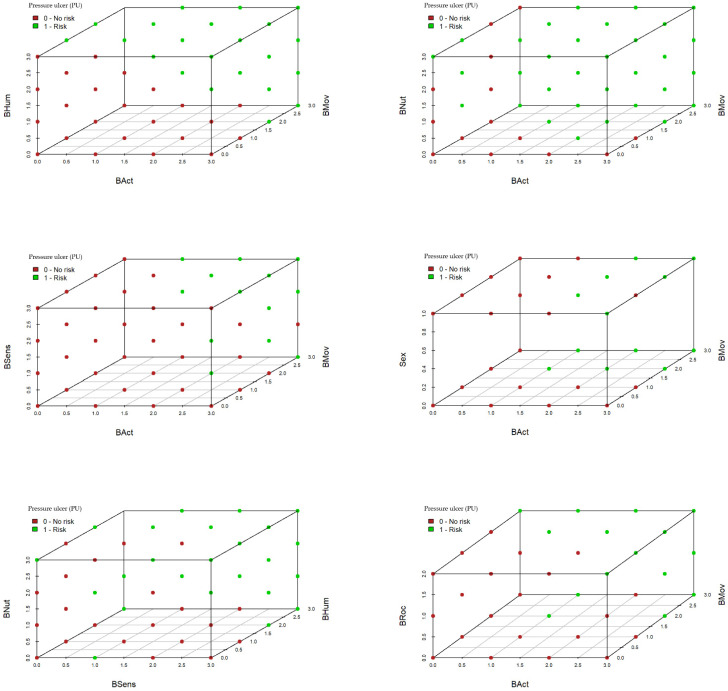
PU risk classification based on three subscales.

The activity (BAct) and mobility (BMov) subscales combined with any one of the remainder of the subscales or the variable sex could provide adequate classifiers. Figure 3 shows six 3D scatterplots where this fact can be analyzed. Considering Figure 3 as a matrix of graphical outputs with three rows and two columns, it is immediately clear that the scatterplot in position (row = 1, column = 1) is the best concerning the adequate classification in one PU risk level or another, because the green and red dots are completely separated. This plot corresponds to the joint classification of the activity, mobility, and skin moisture (BHum) subscales.

### 3.3. Explanatory Model for Pressure Ulcer Risk Prognosis Based on the Six Braden Subscales

In this section a binary-response logistic regression model for PU risk classification is performed based on the variable sex and the six Braden subscales. The stepwise forward–backward selection model included the six Braden subscales in the binary logistic regression model as relevant for the prognosis of patients’ pressure ulcer risk. The estimated model for risk prognosis has the following form:

$${\hat{L}}_{i,j,k,l,m,n}={\hat{B}}_{0}+{\hat{B}}_{BAct}{\left(BAct\right)}_{i}+\;{\hat{B}}_{BMov}{\left(BMov\right)}_{j}+{\hat{B}}_{BRoc}{\left(BRoc\right)}_{k}+{\hat{B}}_{BSens}{\left(BSens\right)}_{l}+\;{\hat{B}}_{BHum}{\left(BHum\right)}_{m}+{\hat{B}}_{BNut}{\left(BNut\right)}_{n}$$

i,j,l,m,n=0,1,2,3/k=0,1,2

$${\left(\mathrm{BAct}\right)}_{0}={\left(\mathrm{BMov}\right)}_{0}={\left(\mathrm{BRoc}\right)}_{0}={\left(\mathrm{BSens}\right)}_{0}={\left(\mathrm{BHum}\right)}_{0}={\left(\mathrm{BNut}\right)}_{0}=0$$

The parameters estimated for each subscale in the binary logistic regression model can be found in Table 2 below.

The chi-square log-likelihood test for this model was X^2^(8, N = 16,215) = 2538.20, *p* < 0.001. Therefore, when these variables were included in the model, the fit improved significantly compared to a model than only takes the constant into account. The Stukel goodness-of-fit test for this model was X^2^(2, N = 16,215) = 15,063, *p* < 0.001. These results did not conclude, therefore, that the model produce a good fit at population level for the risk of developing pressure ulcer.

In light of the results of the *z*-test (see Table 2), all the levels of the subscales are significant at a population-based level (*p* < 0.001). The prognosis change ratio for the levels considered (no risk vs. risk of development pressure ulcer) was analysed for all the explanatory variables with respect to the baseline category. For instance, with regard to the activity scale, it should be noted that the odds of PU development for bedridden patients is 69-fold the odds of those who frequently wander (OR = 68,871.66; 95% CI: 25,591.10–194,852.80), 742 times in a patient who chair wanders (OR = 742.48; 95% CI: 454.86–2344.90) and 16 times in a patient that occasionally wanders (OR = 16.61; 95% CI: 7.92–36.60). As to the scale of mobility is concerned, the advantage of PU development was 14,913 times in completely limited patients than in those with no limitation (OR = 14,913.17; 95% CI: 3041.18–87,553.03), 295 times in patients very limited (OR 295.89; 95% CI: 70.81–1510.20) and 9 times for slightly patients (OR = 9.68; 95% CI: 2.32–48.91). For the shearing scale, the prognosis ratio for PU risk is multiplied by 1236 if the patient has problems with respect to that with no problems (OR = 1236.45; 95% CI: 742.48–2100.65), and is 21-fold if the patient has potential problems (OR = 21.33; 95% CI: 14.59–31.50). The relevant prognosis ratios for the scales related to sensibility, skin moisture and nutritional status can be also immediately identified in the table above.

**Figure 4:**
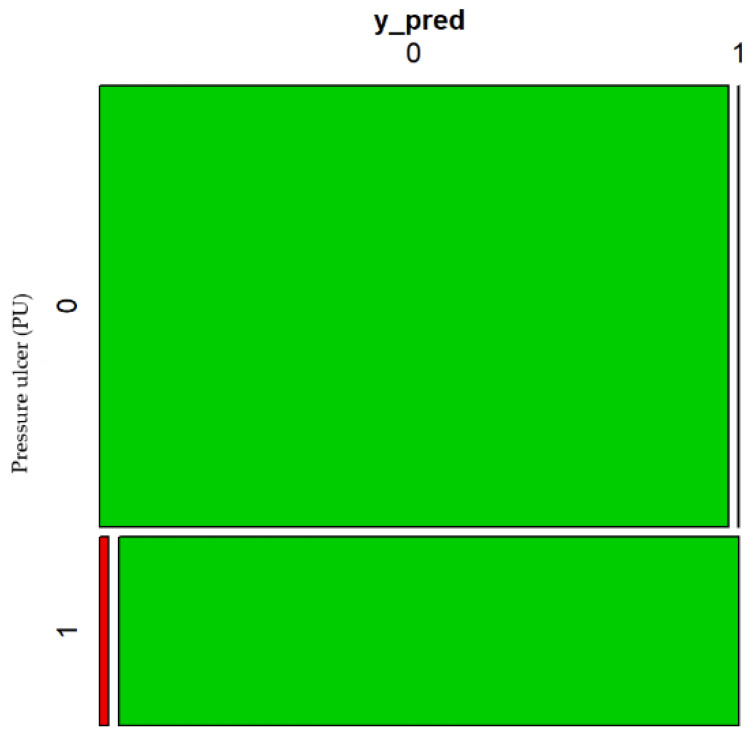
Confusion matrix 1 of the estimated model.

Finally, it is worth mentioning that this model has a rate of correct classifications of 99.5%. Figure 4 shows the confusion matrix of the model supporting this fact.

**Figure 5:**
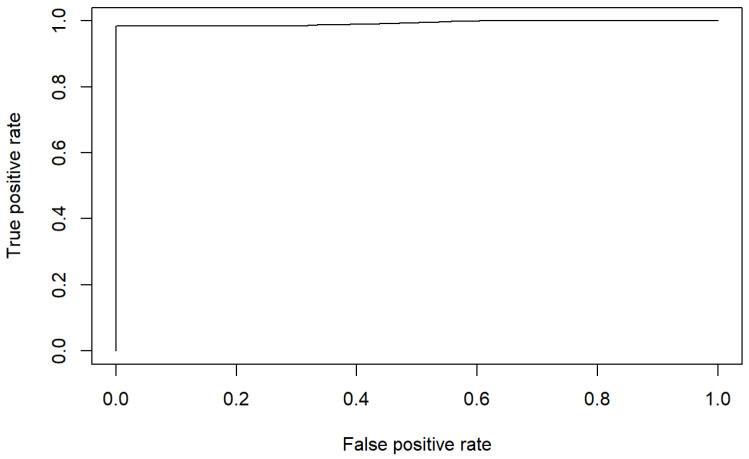
ROC 1 of the estimated model.

In addition, the area under the ROC is of 99.31% which confirms the good discrimination ability of the model for PU risk identification. Figure 5 shows the ROC curve supporting this fact.

Finally, the values of the parameters of internal validity given by the sensitivity (S) and specificity (SP), and the safety indices given by the positive predictive value (PPV) and the negative predictive value (NPV), are listed below:
S = 99.93%PPV = 99.36%SP = 98.50%NPV = 99.83%

Therefore, this model produces the same classification as the Braden Scale with high accuracy.

### 3.4. Explanatory Model for Risk Pressure Ulcer Prognosis Based on Activity and Mobility Subscales

The estimated model for risk prognosis based on the activity and mobility subscales has the following form (Table 3 bellow includes the estimated parameters for this model):

$${\hat{L}}_{i,j}={\hat{B}}_{0}+{\hat{B}}_{\mathrm{BAct}}{\left(\mathrm{BAct}\right)}_{i}+{\hat{B}}_{\mathrm{BMov}}{\left(\mathrm{BMov}\right)}_{j};\;i,j=0,1,2,3;\;{\left(\mathrm{BAct}\right)}_{0}={\left(\mathrm{BMov}\right)}_{0}=0$$

The Chi-square log-likelihood test for this model was X^2^(8, N = 16,215) = 10,259.70, *p* < 0.001. Therefore, when these variables were included in the model, the fit improved significantly compared to a model than only takes the constant into account. The Stukel goodness-of-fit test for this model was X^2^(2, N = 16,215) = 2.90, *p* = 0.234 > 0.05. These results point out that the model produces a good fit, at the population level, for the prediction of PU risk.

Once again, in light of the results of the *z*-test (see Table 3), all the levels of the subscales are significant at a population-based level (*p* < 0.001). For the activity scale, it should be noted that the prognosis ratio of PU development for b patients is 177 times that in those who frequently wander (OR = 177.68; 95% CI: 117.92–275.89), 23 times higher for patients in the chair-wandering category (OR = 23.34; 95% CI: 16.44–34.47), and 4 times higher for patients who occasionally wander (OR = 4.14; 95% CI: 2.89–6.05). As regards the scale of mobility, the advantage of developing a pressure ulcer was 148-fold in completely limited patients when compared to those with no limitation (OR = 148.41; 95% CI: 70.11–368.71), 30 times higher in very limited patients (OR 29.96; 95% CI: 14.73–72.24), and 3 times higher in slightly limited patients (OR = 2.97; 95% CI: 1.46–7.24).

**Figure 6:**
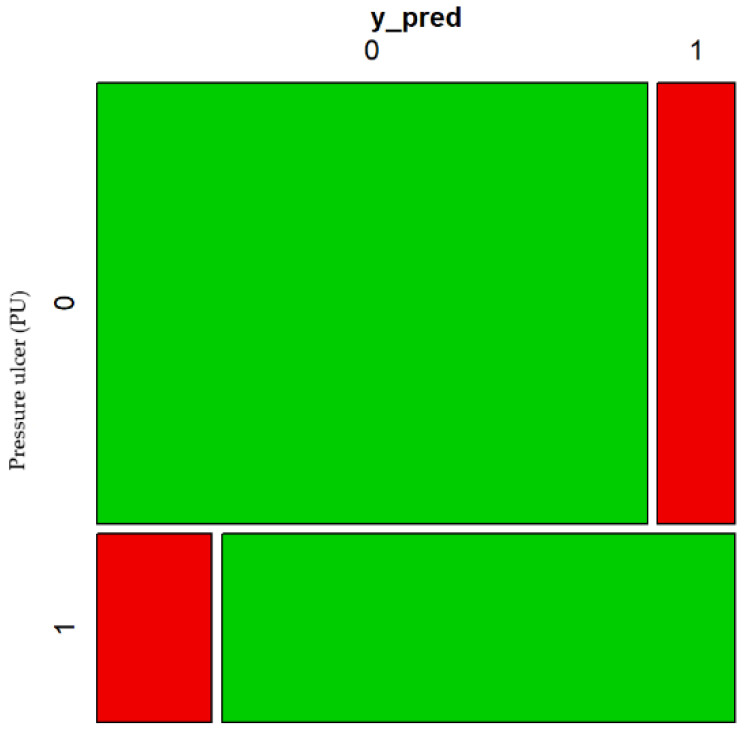
Confusion matrix 2 of the estimated model.

As for the previous model, it is relevant to mention that this model has a rate of correct classifications of 85.8%. Figure 6 shows the confusion matrix supporting this fact.

**Figure 7:**
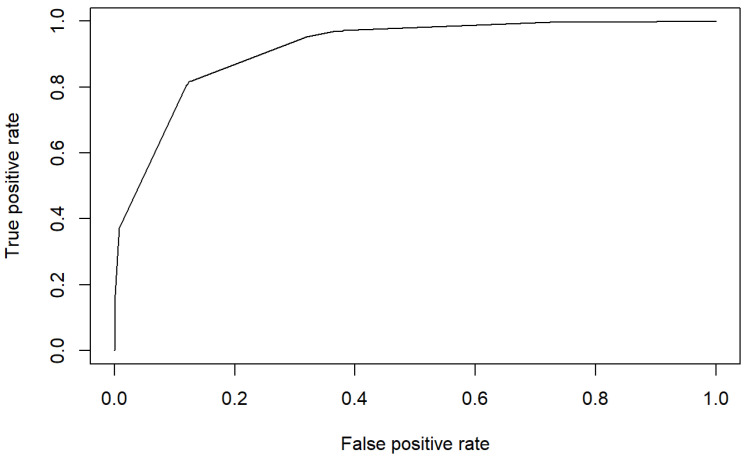
ROC 2 of the estimated model.

In addition, the area under the ROC curve is of 91.68% which confirms the good discrimination of the model to identify PU risk using only these two subscales. Figure 7 shows the ROC curve supporting this fact.

Finally, the values of the sensitivity (S) and specificity I, as well as the positive predictive value (PPV) and the negative predictive value (NPV), are listed below:
S = 87.57%PPV = 91.78%SP = 81.69%NPV = 73.78%

### 3.5. Explanatory Model for Pressure Ulcer Risk Prognosis Based on Activity, Mobility, and Skin Moisture Subscales

The estimated model for risk prognosis bases on the activity, mobility, and skin moisture subscales has the following form (Table 4 bellow includes the estimated parameters for this model):

$${\hat{L}}_{i,j,k}={\hat{B}}_{0}+{\hat{B}}_{\mathrm{BAct}}{\left(\mathrm{BAct}\right)}_{i}+{\hat{B}}_{\mathrm{BMov}}{\left(\mathrm{BMov}\right)}_{j}+{\hat{B}}_{\mathrm{BHum}}I;\;i,j=0,1,2,3;\;{\left(\mathrm{BAct}\right)}_{0}={\left(\mathrm{BMov}\right)}_{0}={\left(\mathrm{BHum}\right)}_{0}=0$$

The chi-square log-likelihood test for this model was X^2^(8, N = 16,215) = 7207.69, *p* < 0.001. Therefore, when these variables were included in the model, the fit improved significantly compared to a model than only takes the constant into account. The Stukel goodness-of-fit test for this model was X^2^(2, N = 16,215) = 33.54, *p* < 0.001. These results concluded, therefore, that the model did not produce a good fit at population level with the risk of developing pressure ulcer.

Once again, in light of the results of the *z*-test (see Table 3), all the levels of the subscales are significant at a population-based level (*p* < 0.001 or *p* < 0.05). For the activity scale, it should be note that the prognosis ratio of PU development for bedridden patients is 190-fold that of patients who frequently wander (OR = 190.57; 95% CI: 79.04–533.79), 30 times higher for patients in the chair-wandering category (OR = 29.96; 95% CI: 13.20–80.64), and 3 times higher for those who occasionally wander (OR = 2.72; 95% CI: 1.20–7.32). As regards the scale of mobility, the advantage of developing a pressure ulcer was 190-fold in completely limited patients when compared to those with no limitation (OR = 190.57; 95% CI: 117.92–314.19), 18 times higher in very limited patients (OR 17.64; 95% CI: 11.70–27.39), and 3 times higher in slightly limited patients (OR = 3.16; 95% CI: 2.12–4.90). For the skin moisture scale, the prognosis ratio for risk of pressure ulcer is multiplied by 450 if the patient is constantly wet, when compared to those with no problems of skin moisture (OR = 450.34; 95% CI: 304.90–678.58), the risk is multiplied by 79 if the patient is often web (OR = 79.04; 95% CI: 63.43–99.48), and by 10 times if the patient is occasionally wet (OR = 10.28; 95% CI: 8.50–12.68).

**Figure 8:**
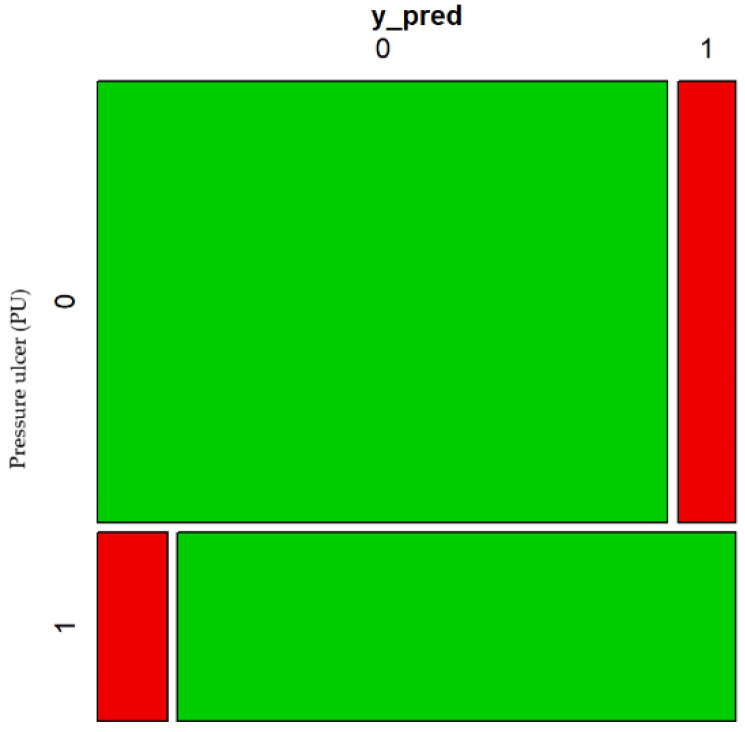
Confusion matrix 3 of the estimated model.

As for the previous model, is relevant to mention that this model has a rate of correct classifications of 90.2%. Figure 8 shows the confusion matrix of this model supporting this fact.

**Figure 9:**
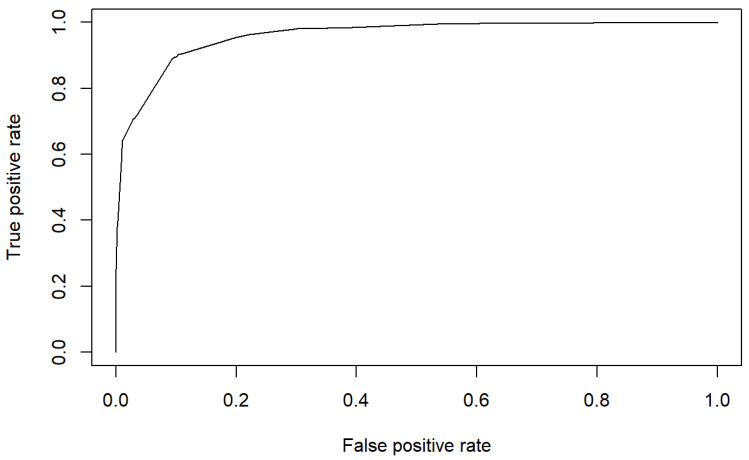
ROC 3 of the estimated model.

In addition, the area under the ROC is of 96.06%, which confirms the good discrimination of the model to identify risk or absence of risk regarding pressure ulcers with this subscale only. Figure 9 shows the ROC curve supporting this fact.

Finally, the values of the internal validity parameters and the safety indices are listed below:
S = 90.74%PPV = 95.00%SP = 88.83%NPV = 80.42%

## 4. Discussion

This work aimed at identifying groups of Braden subscales that provide efficient classification models, and at quantifying the effect of each subscale within the model for prognosis at a level of worsening risk of developing pressure ulcers for immobilized patients. With regard to the first objective and the Braden subscales as explanatory variables, three models that provide a first approximation of level changes in the risk of developing pressure ulcers were obtained. These models included all the Braden subscales considered as relevant to fit them. They are able to predict the probability of an individual being at risk of developing a pressure ulcer. The validation of the model including all the scales showed the same behaviour as the Braden Scale for pressure ulcers risk assessment. The mobility and activity subscales are relevant risk factors involving increasing risk of pressure ulcers. Indeed, these two scales provide a reliable model for risk classification due to the high values of the different internal validity parameters and safety indices. The mobility, activity, and skin moisture scales, jointly, even improve the reliability due to higher values of these parameters. With regard to the second objective, the result showed that high levels of all the scales were associated to situations of greater risk of a pressure ulcer developing. It is relevant to highlight the mobility and activity subscales that were associated to these risk situations for all the models. The Braden’s mobility subscale is considered as an independent risk factor for PU development [21,22]. The activity subscale is considered with an overall pooled effect in [23]. However, the subscale skin moisture is also considered in [23] but it did not reach significance. Mobility and activity, jointly, are considered in [16] with patients residing in the long-term care. According to the results of this study, immobilized patients characterized by high levels of activity and mobility, jointly, understanding this fact as a higher limitation, suffer greater risk of developing pressure ulcers.

After the results obtained in this work, regarding the validity of the mobility and activity subscales of the Braden Scale as predictors of pressure ulcer risk, it is reasonable to consider how professionals make decisions using a methodology that adds little value, versus other simpler and more efficient ones, that involved the nurse as an expert on the patient [24,25]. In this sense, a change in practice could be proposed using these subscales due to their high predictive value to identify the risk of pressure ulcers quickly and efficiently [1]. Fast decision-making results in the implementation of an adequate care plan from practically the first signs of risk of developing pressure ulcers. Consequently, the complete Braden Scale could ultimately be used for the categorization and origin of risk as an essential tool for organizing the different resources for patients.

Finally, it is very important to encourage nursing professionals to use their experience to assess the risk of developing pressure ulcers in these patients in order to develop strategies for PU prevention and thus reduce the health burden associated with pressure ulcers.

### Clinical Implications

Nurses are essential in the early care of patients. This is the reason for a great demand for care that overloads professionals with work. On the other hand, PUs affect the quality of life of patients and their families. This study provides nurses with confidence in their professional criteria based on the mobility and activity subscales, jointly. This evidence-based practice can be improved with the skin moisture subscale, along with the previous ones. In addition, the six subscales can be also considered by means of a quick-evaluation model for risk assessment of developing pressure ulcers.

## 5. Conclusions

Immobilized patients are at greater risk of PU incidence. This represents a health problem with a significant impact in the quality of life of affected people and their families. Therefore, quick decision making by health care professionals becomes paramount for the application of preventive measures. In this sense, the objective nursing criteria is an essential addition. The SIRUPP study provides relevant information to ensure the trustworthiness of a diagnosis of PU risk based only in the objective experience of the health professional.

## Figures and Tables

**Table 1:** Description of variables.

Response Variable	Level	% (N)
UP = Pressure ulcer (N = 16,215)	(0) No risk	70.0 (11,354)
	(1) Risk	30.0 (4861)
Explantatory variables	Level	% (N)
BSens = Sensory perception (N = 16,215) (Ability to respond meaningfully to pressure-related discomfort)	(0) No impairment	23.2 (3754)
	(1) Slightly limited	43.6 (7064)
	(2) Very limited	25.7 (4179)
	(3) Completely limited	7.5 (1218)
BHum = Skin moisture (N = 16,215) (Degree to which skin is exposed to moisture)	(0) Rarely moist	39.3 (6371)
	(1) Occasionally moist	37.9 (6147)
	(2) Often moist	17.0 (2762)
	(3) Constantly wet	5.8 (935)
BAct = Activity (N = 16,215) (Degree of physical activity)	(0) Walks frequently	19.5 (3158)
	(1) Walks occasionally	44.0 (7142)
	(2) Chairfast	26.2 (4249)
	(3) Bedfast	10.3 (1666)
BMov = Mobility (N = 16,215) (Ability to change and control body position)	(0) No limitations	8.4 (1357)
	(1) Slightly limited	39.8 (6458)
	(2) Very limited	44.3 (7191)
	(3) Completely immobile	7.5 (1209)
BNut = Nutritional status (N = 16,215) (Usual food intake pattern)	(0) Excellent	7.6 (1240)
	(1) Adequate	74.1 (12,023)
	(2) Probably inadequate	15.2 (2470)
	(3) Very poor	3.1 (482)
Broc = Shearing (N = 16,215) (Friction and shear)	(0) No apparent problem	34.9 (5659)
	(1) Potential problem	50.1 (8128)
	(2) Problem	15.0 (2428)
S = Sex (N = 16,215)	(0) Female	69.8 (11,323)
	(1) Male	30.2 (4892)

**Table 2:** Prognosis model for pressure ulcer (PU).

Subscale	B	DT	Z	*p*	OR	CI for 95% OR	
						Lower	Upper
Constant	−29.53	1.16	−25.48	<0.001			
${\left(\mathrm{BAct}\right)}_{1}$	2.81	0.39	7.24	<0.001	16.61	7.92	36.60
${\left(\mathrm{BAct}\right)}_{2}$	6.91	0.42	16.58	<0.001	742.48	454.86	2344.90
${\left(\mathrm{BAct}\right)}_{3}$	11.14	0.52	21.53	<0.001	68,871.66	25,591.10	194,852.86
${\left(\mathrm{BMov}\right)}_{1}$	2.27	0.77	2.95	<0.001	9.68	2.32	48.91
${\left(\mathrm{BMov}\right)}_{2}$	5.69	0.77	7.39	<0.001	295.89	70.81	1510.20
${\left(\mathrm{BMov}\right)}_{3}$	9.61	0.85	11.32	<0.001	14,913.17	3041.18	87,553.03
${\left(\mathrm{BRoc}\right)}_{1}$	3.06	0.20	15.59	<0.001	21.33	14.59	31.50
${\left(\mathrm{BRoc}\right)}_{2}$	7.12	0.27	26.75	<0.001	1236.45	742.48	2100.65
${\left(\mathrm{BSens}\right)}_{1}$	3.93	0.24	16.26	<0.001	50.91	32.14	83.10
${\left(\mathrm{BSens}\right)}_{2}$	7.93	0.28	28.04	<0.001	2779.43	1619.71	4914.77
${\left(\mathrm{BSens}\right)}_{3}$	11.54	0.44	26.19	<0.001	102,744.44	44,355.86	250,196.03
${\left(\mathrm{BHum}\right)}_{1}$	3.82	0.19	19.88	<0.001	45.60	31.82	67.36
${\left(\mathrm{BHum}\right)}_{2}$	7.89	0.25	31.05	<0.001	2670.44	1635.98	4402.82
${\left(\mathrm{BHum}\right)}_{3}$	11.67	0.42	27.49	<0.001	117,008.28	52,052.08	273,758.06
${\left(\mathrm{BNut}\right)}_{1}$	4.09	0.32	12.73	<0.001	59.74	32.14	113.30
${\left(\mathrm{BNut}\right)}_{2}$	8.02	0.38	21.34	<0.001	3041.18	1480.30	6438.17
${\left(\mathrm{BNut}\right)}_{3}$	11.68	0.60	19.40	<0.001	118,184.24	37,049.12	388,481.18

Note: BAct = activity, BMov = mobility, BRoc = shearing, BSens = sensibility, BHum = skin moisture, BNut = nutritional status, B = estimated parameter, DT = standard deviation, Z = Z statistic, *p* = *p*-value, OR = odds ratio, CI = confidence interval, Lower = lower limit of the CI, Upper = upper limit of the CI.

**Table 3:** Prognosis model for pressure ulcer (PU) based on activity and mobility subscales.

Subscale	B	DT	Z	*p*	OR	CI for 95% OR	
						Lower	Upper
Constant	−6.04	0.41	−14.89	<0.001			
${\left(\mathrm{BAct}\right)}_{1}$	1.42	0.19	7.51	<0.001	4.14	2.89	6.05
${\left(\mathrm{BAct}\right)}_{2}$	3.15	0.19	16.73	<0.001	23.34	16.44	34.47
${\left(\mathrm{BAct}\right)}_{3}$	5.18	0.22	24.07	<0.001	177.68	117.92	275.89
${\left(\mathrm{BMov}\right)}_{1}$	1.09	0.40	2.72	<0.001	2.97	1.46	7.24
${\left(\mathrm{BMov}\right)}_{2}$	3.40	0.40	8.54	<0.001	29.96	14.73	72.24
${\left(\mathrm{BMov}\right)}_{3}$	5.00	0.42	12.01	<0.001	148.41	70.11	368.71

Note: BAct = Activity, BMov = Mobility, B = estimated parameter, DT = standard deviation, Z = Z statistic, *p* = *p*-value, OR = odds ratio, CI = confidence interval, Lower = lower limit of the CI, Upper = upper limit of the CI.

**Table 4:** Prognosis model for pressure ulcer (PU) based on activity, mobility, and skin moisture subscales.

Subscale	B	DT	Z	*p*	OR	CI for 95% OR	
						Lower	Upper
Constant	−8.37	0.49	−17.25	<0.001			
${\left(\mathrm{BAct}\right)}_{1}$	1.00	0.46	2.20	<0.05	2.72	1.20	7.32
${\left(\mathrm{BAct}\right)}_{2}$	3.40	0.46	7.47	<0.001	29.96	13.20	80.64
${\left(\mathrm{BAct}\right)}_{3}$	5.25	0.48	10.90	<0.001	190.57	79.04	533.79
${\left(\mathrm{BMov}\right)}_{1}$	1.15	0.21	5.39	<0.001	3.16	2.12	4.90
${\left(\mathrm{BMov}\right)}_{2}$	2.87	0.22	13.34	<0.001	17.64	11.70	27.39
${\left(\mathrm{BMov}\right)}_{3}$	5.25	0.25	20.98	<0.001	190.57	117.92	314.19
${\left(\mathrm{BHum}\right)}_{1}$	2.33	0.10	22.64	<0.001	10.28	8.50	12.68
${\left(\mathrm{BHum}\right)}_{2}$	4.37	0.12	37.79	<0.001	79.04	63.43	99.48
${\left(\mathrm{BHum}\right)}_{3}$	6.11	0.20	29.89	<0.001	450.34	304.90	678.58

Note: BAct = activity, BMov = mobility, BHum = skin moisture, B = estimated parameter, DT = standard deviation, Z = Z statistic, *p* = *p*-value, OR = odds ratio, CI = confidence interval, Lower = lower limit of the CI, Upper = upper limit of the CI.

## Data Availability

Data available upon request to the authors.
